# SelO‐mediated nicotinamide adenine dinucleotide hydrolysis and its implications for metabolic therapies

**DOI:** 10.1002/ctm2.70773

**Published:** 2026-08-05

**Authors:** Teng Zhang, Ting Wang, Yuan Fu

**Affiliations:** ^1^ The Sichuan Provincial Key Laboratory for Genetic Diseases Institute for Laboratory Medicine Sichuan Provincial People's Hospital School of Medicine University of Electronic Science and Technology of China Chengdu China; ^2^ Department of Pharmacology and Tianjin Key Laboratory of Inflammatory Biology School of Basic Medical Sciences Tianjin Medical University Tianjin China

1

Nicotinamide adenine dinucleotide (NAD) exists in two distinct cellular forms: the oxidised state NAD+ and the reduced state NADH. Acting as a dynamic redox intermediary within cellular energy production, this molecule drives essential pathways, including the tricarboxylic acid (TCA) cycle for pyruvate metabolism (derived from glycolysis) and β‐oxidation for lipid catabolism. Essentially, NAD+ accepts two electrons and one proton from carbohydrates and lipids, the primary energy substrates for mammalian cells, to become the high‐energy carrier NADH. NADH subsequently fuels oxidative phosphorylation to generate ATP. Consequently, the NAD+/NADH ratio acts as a master regulator of nearly all metabolic and oxidoreductive processes, including glycolysis and fatty acid oxidation.^1^, ^2^

However, the regulation of NAD extends beyond toggling between NAD+ and NADH. The absolute size of the overall NAD pool is a critical and independent variable. Historically, much of the research has relied solely on measuring the NAD+/NADH ratio, operating under the assumption that fluctuations in this parameter adequately reflect the availability of these molecules for metabolic reactions. In reality, mammalian cells actively acquire NAD through biosynthesis from precursors like nicotinamide and consume it via enzymes such as PARPs and sirtuins (SIRTs) during post‐translational protein modifications.^1^, ^3^ Conceivably, the absolute size of the NAD pool should also dictate its molecular availability. Furthermore, NAD pools are highly compartmentalised within the cell. The extracellular, cytosolic, and mitochondrial spaces represent three relatively independent NAD pools. Specifically, the shuttling of NAD between the cytosol and mitochondria does not simply occur via passive diffusion, but requires the dedicated transporter SLC25A51.^4^

Although the biosynthesis of NAD is well characterised in both the mitochondrial matrix and the cytosol, how NAD levels are proactively controlled through degradation within the mitochondria remains poorly understood. We recently identified that the mitochondrial matrix protein SELENOO (SelO), a selenocysteine‐containing protein, catalyses the hydrolysis of NAD into nicotinamide mononucleotide (NMN) and AMP.^5^ Interestingly, this enzyme was originally characterised as mediating AMPylation, a rare post‐translational modification that transfers an AMP moiety from ATP to serine, threonine, or tyrosine residues on target proteins.^6^ While both enzymatic functions share the same adenosine‐binding pocket, they differ in their reliance on the C‐terminal selenocysteine. Specifically, this rare residue is absolutely critical for SelO's NAD hydrolysis activity but remains dispensable for its AMPylation function. Physiologically, cells may harbour both the full‐length, selenocysteine‐containing isoform and a truncated isoform resulting from translational termination at the selenocysteine site, with the balance dictated by environmental selenium availability.^7^

Beyond its AMPylation function, we demonstrated that full‐length SelO provides an indispensable and localised NAD degradation mechanism within the mitochondrial matrix. This hydrolytic activity is triggered by an elevated matrix pH, positioning SelO as a responsive safety valve against sustained mitochondrial overactivation. Furthermore, SelO directly associates with the mitochondrial trifunctional protein complex (HADHA/HADHB) to selectively suppress lipid utilisation, a process that strictly requires NAD as an intermediate energy carrier (Figure 1).

**FIGURE 1:**
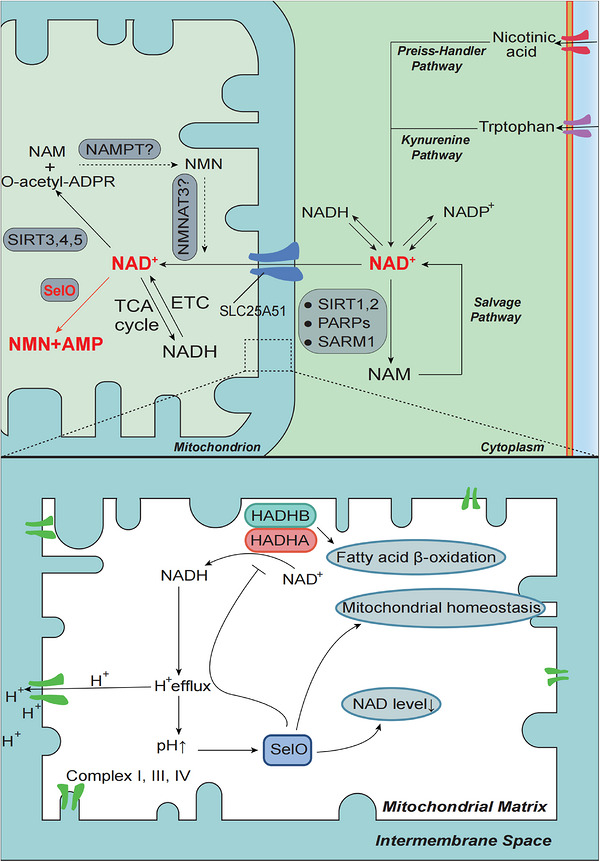
Schematic representation of cellular nicotinamide adenine dinucleotide (NAD) compartmentalisation and its regulation by SelO. The top panel depicts the key processes and enzymes governing the cytosolic and mitochondrial NAD pools. The bottom panel illustrates how SelO maintains mitochondrial homeostasis and regulates fatty acid β‐oxidation through the localised hydrolysis of NAD.

Understanding the function of SelO sheds light on important pharmacological insights. First and foremost, the current clinical landscape is dominated by an enthusiasm for NAD precursors, driven by the premise that augmenting intracellular NAD pools universally restores metabolic vitality and combats age‐related decline.^8^, ^9^ Precursors such as nicotinamide and nicotinamide riboside are widely marketed worldwide as anti‐ageing nutritional supplements and sold on shelves in pharmacies. However, this “more is better” paradigm overlooks a fundamental principle of cellular homeostasis: the precise spatiotemporal regulation of metabolite pools. By identifying SelO as a localised enzyme that directly hydrolyses mitochondrial NAD in response to metabolic stress, we have uncovered a critical safety valve that protects against mitochondrial overactivation. For clinicians and translational researchers, this underscores a vital shift in perspective: the unchecked expansion of the NAD pool is not inherently benign, and future therapeutic interventions must account for the strict subcellular compartmentation.

Second, we demonstrated that SelO locally degrades NAD to specifically suppress fatty acid β‐oxidation, effectively putting the brakes on lipid utilisation during periods of metabolic overactivation. While accelerating lipid catabolism may appear beneficial for the treatment of hepatic steatosis or metabolic syndrome, disabling the SelO “brake” results in severe mitochondrial fragmentation, membrane permeabilisation, and subsequent tissue inflammation, as evidenced by elevated AST and ALT levels in knockout models. Thus, the uncontrolled acceleration of lipid catabolism is not without pathological consequence, highlighting the need for highly targeted metabolic interventions rather than systemic overload.

In sum, these findings challenge the prevailing clinical assumption that systemic NAD supplementation is a universally beneficial intervention. The mitochondrial NAD pool is actively safeguarded by SelO to prevent metabolic overloading. Ultimately, true metabolic health should rely on tightly compartmentalised control of metabolism.

## AUTHOR CONTRIBUTIONS

Teng Zhang and Yuan Fu conceived the idea. Teng Zhang prepared the figure. Yuan Fu wrote the manuscript with editorial contribution from Teng Zhang and Ting Wang.

## CONFLICT OF INTEREST STATEMENT

The authors declare no conflict of interest.

## ETHICS STATEMENT

Not applicable. This article does not involve any new studies with human participants or animal subjects.
